# CR-LAAO, an L-amino acid oxidase from *Calloselasma rhodostoma* venom, as a potential tool for developing novel immunotherapeutic strategies against cancer

**DOI:** 10.1038/srep42673

**Published:** 2017-02-16

**Authors:** Tássia R. Costa, Danilo L. Menaldo, Karina F. Zoccal, Sandra M. Burin, Alexandre F. Aissa, Fabíola A. de Castro, Lúcia H. Faccioli, Lusânia M. Greggi Antunes, Suely V. Sampaio

**Affiliations:** 1 Department of Clinical Analyses, Toxicology and Food Sciences, School of Pharmaceutical Sciences of Ribeirão Preto, University of São Paulo, FCFRP-USP, Ribeirão Preto, SP, Brazil

## Abstract

L-amino acid oxidases from snake venoms have been described to possess various biological functions. In this study, we investigated the inflammatory responses induced *in vivo* and *in vitro* by CR-LAAO, an L-amino acid oxidase isolated from *Calloselasma rhodostoma* venom, and its antitumor potential. CR-LAAO induced acute inflammatory responses *in vivo*, with recruitment of neutrophils and release of IL-6, IL-1β, LTB_4_ and PGE_2_. *In vitro*, IL-6 and IL-1β production by peritoneal macrophages stimulated with CR-LAAO was dependent of the activation of the Toll-like receptors TLR2 and TLR4. In addition, CR-LAAO promoted apoptosis of HL-60 and HepG2 tumor cells mediated by the release of hydrogen peroxide and activation of immune cells, resulting in oxidative stress and production of IL-6 and IL-1β that triggered a series of events, such as activation of caspase 8, 9 and 3, and the expression of the pro-apoptotic gene *BAX*. We also observed that CR-LAAO modulated the cell cycle of these tumor cells, promoting delay in the G0/G1 and S phases. Taken together, our results suggest that CR-LAAO could serve as a potential tool for the development of novel immunotherapeutic strategies against cancer, since this toxin promoted apoptosis of tumor cells and also activated immune cells against them.

The immune system is able to find and destroy abnormal cells, preventing the development of a variety of illnesses. Recently, numerous medical and scientific studies have been in search of ways to use our own immune system to confront diseases. In this context, new discoveries have led to treatment strategies, known as immunotherapy, with excellent results against cancer^1^,^2^, so much so that the American Society of Clinical Oncology elected immunotherapy as the biggest breakthrough against cancer in 2016^3^.

Current immunotherapy used in cancer treatment has been developed in two main areas: the use of antibodies, which are capable of specifically recognizing tumor cells and redirecting an immune response against them, e.g. Antibody-Dependent Cell-Mediated Cytotoxicity (ADCC) and Complement Dependent Cytotoxicity (CDC), and the application of immunomodulatory cytokines^4^.

Cytokines are a large family of mainly soluble proteins and glycoproteins that function as key modulators of the immune system. They include interleukins (ILs), interferons (IFNs), growth factors, colony stimulating factors (CSFs), the tumor necrosis factor (TNF) family and chemokines. Cytokines activate receptors on target cells, thus their use in cancer therapy require that they reach tumor cells in order to exert their immunomodulating and cytotoxic effects^4^. Considering this, pro- and anti-inflammatory cytokines have been used in the last few years as immunotherapy agents in the treatment of metastatic cancer and autoimmune diseases^5^. The pro-inflammatory cytokine IL-2 (Proleukin^®^), for example, is current used in the treatment of metastatic melanoma and renal cell carcinoma, inducing the apoptosis of tumor cells by activating cytotoxic immune cells, such as natural killers and macrophages^5^.

Despite the potential of immunotherapy in cancer treatment, these strategies have to face several challenges in order to be effective, which includes the fact that tumor cells present many mutations that inhibit detection and destruction by the immune system. Thus, the investigation of novel antitumor agents, including those from natural sources, is of utmost importance for the development of new strategies for addressing these problems.

Snake venoms are rich sources of bioactive proteins, many of which have enzymatic activity and are responsible for several biological effects^6^,^7^. Some of these toxins have been shown to present great potential as drugs for treating different conditions, e.g. L-amino acid oxidases (LAAOs), which have the ability to destroy various types of tumor cells and microorganisms, such as bacteria and protozoa^8^,^9^,^10^,^11^. Besides, LAAOs have also been investigated regarding their inflammatory potential^12^,^13^.

CR-LAAO, a LAAO from *Calloselasma rhodostoma* snake venom, is one of the most studied toxins from this class of enzymes and already had its structural, molecular and biochemical characteristics unfolded^14^, however, little is known about its pharmacological activities. Recently, our research group highlighted some of its biological effects, including bactericidal effects against *Staphylococcus aureus* and *Escherichia coli* strains, inhibition of *Candida albicans* growth, and cytotoxic activity on *Leishmania* species and *Trypanosoma cruzi*^11^. CR-LAAO also showed high cytotoxicity on HepG2 and HL-60 tumor cells, with lower effects on human mononuclear cells (PBMC)^11^. In addition, the inflammatory effects of CR-LAAO were assessed on human neutrophils *in vitro*, showing stimulation of chemotaxis and the production of reactive oxygen species (superoxide anion and hydrogen peroxide), pro-inflammatory cytokines (IL-6, IL-8 and TNF-α) and lipid mediators (PGE_2_ and LTB_4_)^13^,^15^.

Therefore, considering that CR-LAAO has been described to promote both antitumor and pro-inflammatory effects *in vitro*, it is possible to suggest that these two effects could be somehow connected. With that in mind, the present study was designed to investigate the pro-inflammatory properties of CR-LAAO *in vivo* and *in vitro*, as well as its apoptotic activity on human tumor cells. The correlation of these results could be valuable to assess potential applications of this enzyme in cancer immunotherapy.

## Results

### CR-LAAO induces neutrophils recruitment and inflammatory mediators

**Figure 1:**
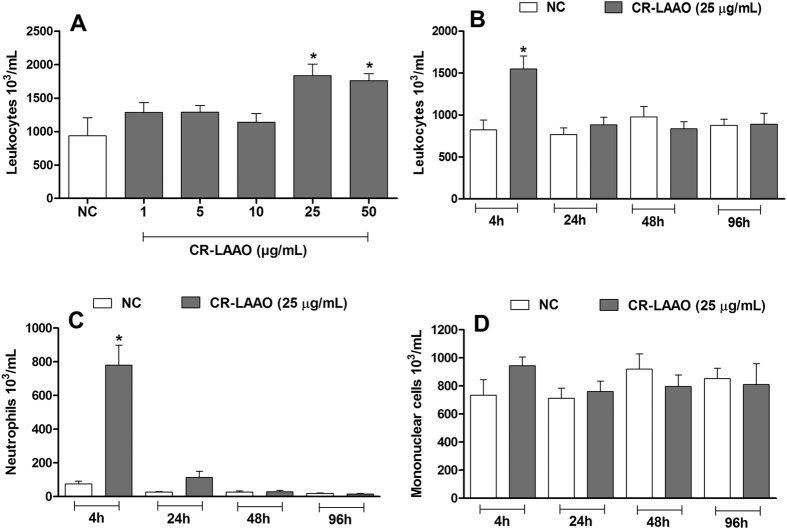
CR-LAAO induces leukocyte recruitment into the peritoneal cavity of mice. Initially, the migration of total leukocytes was evaluated after 4 h of the injection of CR-LAAO (1, 5, 10, 25 or 50 μg/mL) in the peritoneal cavity of mice (**A**). Then, the migration of total leukocytes (**B**), neutrophils (**C**) and mononuclear cells (**D**) was evaluated after 4, 24, 48 and 96 h of the injection of CR-LAAO (25 μg/mL) in the peritoneal cavity of mice. Negative control group (NC) was injected with PBS. Data were expressed as means ± SD (n = 4). *p < 0.05 compared to NC.

Firstly, we investigated the recruitment of cells into the peritoneal cavity of BALB/c mice induced by different concentrations of CR-LAAO (1–50 μg/mL). After 4 hours of treatment, it was observed that only the concentrations of 25 and 50 μg/mL were able to induce the migration of leukocytes into the peritoneal cavity of treated animals (Fig. 1A). Subsequently, the CR-LAAO concentration of 25 μg/mL was used to evaluate the responses induced after different stimulation periods (4, 24, 48 and 96 h). Migration of leukocytes into the peritoneal cavity was observed only after 4 h of administration of the enzyme (Fig. 1B), and differential count of these leukocytes revealed a substantial increase of neutrophils (Fig. 1C) with no significant changes in the number of mononuclear cells compared to the negative control (Fig. 1D).

**Figure 2:**
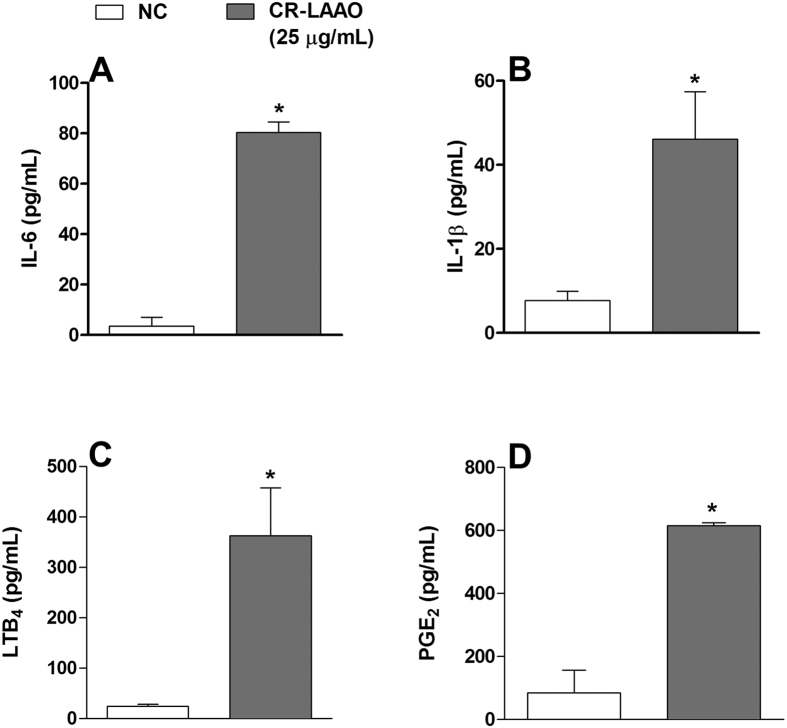
CR-LAAO induces IL-6, IL-1β, PGE_2_ and LTB_4_ production in the peritoneal cavity of mice. Concentrations of the pro-inflammatory cytokines IL-6 (**A**) and IL-1β (**B**), and the lipid mediators LTB_4_ (**C**) and PGE_2_ (**D**) were determined by ELISA in the supernatant of mice peritoneal exudates after 4 h of stimulus with CR-LAAO (25 μg/mL). Negative control group (NC) was injected with PBS. Data were expressed as means ± SD (n = 4). *p < 0.05 compared to NC.

Peritoneal exudate supernatants obtained from mice after 4 h of stimulus with CR-LAAO showed significant increases in the concentrations of IL-6 (Fig. 2A) and IL-1β (Fig. 2B) when compared to the negative control. Nevertheless, no significant levels of the cytokines IL-4, IL-10 and TNF-α were detected (data not shown). There was also a significant increase in the concentrations of the lipid mediators LTB_4_ (Fig. 2C) and PGE_2_ (Fig. 2D) in comparison to the negative control levels. These results indicate that CR-LAAO induced acute inflammatory responses *in vivo* after 4 h of stimulus, with recruitment of neutrophils resulting in increased levels of cytokines, LTB_4_ and PGE_2_.

### CR-LAAO induces IL-6 and IL-1β production via TLR2 and TLR4 dependent signaling

**Figure 3:**
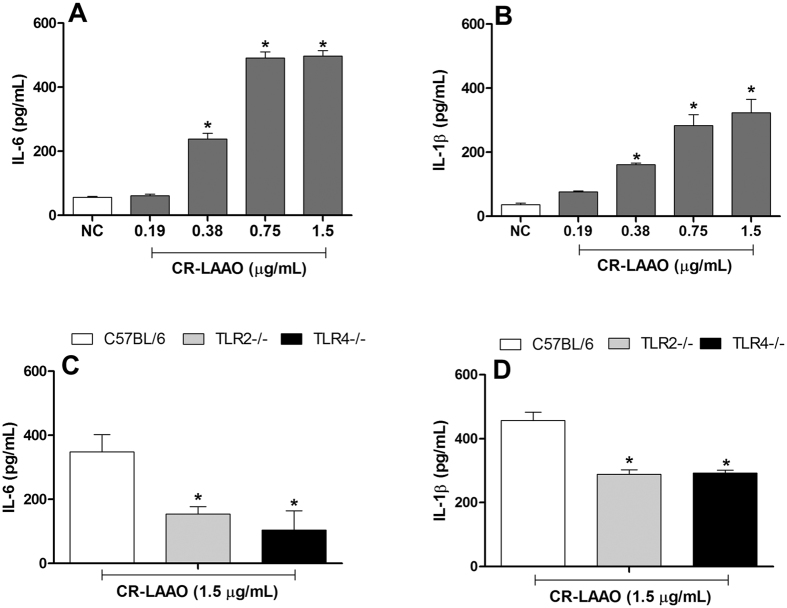
TLR2 and TLR4 mediate the recognition of CR-LAAO and modulate IL-6 and IL-1β production. Peritoneal macrophages from C57BL/6 mice (wild type, TLR2^−/−^ and TLR4^−/−^ knockouts) were stimulated with CR-LAAO (0.19, 0.38, 0.75 or 1.5 μg/mL) for 24 h in a 5% CO_2_ atmosphere at 37 °C. The supernatants were collected and the levels of IL-6 (**A**,**C**) and IL-1β (**B**,**D**) were measured by ELISA. Peritoneal macrophages without stimulation were used as negative controls (NC). Data were expressed as means ± SD (n = 6). *p < 0.05 compared to NC.

After the *in vivo* investigations, the inflammatory effects of CR-LAAO were evaluated *in vitro* on peritoneal macrophages extracted from C57BL/6 mice. After 24 h of stimulus, the toxin (0.38; 0.75 and 1.5 μg/mL) increased the production of IL-6 and IL-1β in comparison to non-stimulated cells (Fig. 3A and B). In order to evaluate if the production of these cytokines was dependent or not of the activation of Toll-like receptors (TLRs), peritoneal macrophages from TLR2^−/−^ and TLR4^−/−^ knockout animals were incubated with CR-LAAO (1.5 μg/mL) for 24 h. We observed that IL-6 (Fig. 3C) and IL-1β (Fig. 3D) concentrations were significantly decreased in the supernatants of TLR2^−/−^ and TLR4^−/−^ peritoneal macrophages when compared to cells from wild type animals. This result suggests that the CR-LAAO-induced production of IL-6 and IL-1β *in vitro* is dependent of the activation of TLR2 and TLR4.

### CR-LAAO-stimulated tumor cell lines produce inflammatory cytokines

**Figure 4:**
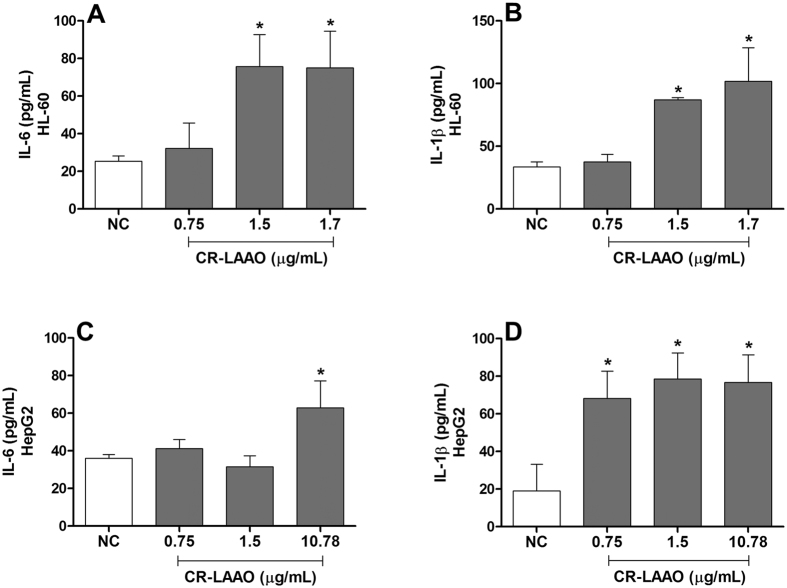
CR-LAAO induces IL-6 and IL-1β production in tumor cells. HL-60 and HepG2 tumor cells (2 × 10^5^ cells) were stimulated with CR-LAAO (0.75, 1.5; 1.7 or 10.78 μg/mL) for 6 h in a 5% CO_2_ atmosphere at 37 °C. The supernatants were collected and the levels of IL-6 (**A**,**C**) and IL-1β (**B**,**D**) were measured by ELISA. Tumor cells without stimulation were used as negative controls (NC). Data were expressed as means ± SD (n = 6). *p < 0.05 compared to NC.

Considering our previous findings about the cytotoxic activity of CR-LAAO on HL-60 (IC_50_ = 1.7 μg/mL) and HepG2 (IC_50_ = 10.78 μg/mL) tumor cells^11^, here we determined the levels of inflammatory cytokines in the cell supernatant after 6 h of treatment with different concentrations of the enzyme (0.75; 1.5; 1.7 or 10.78 μg/mL). HL-60 cells released increased IL-6 (Fig. 4A) and IL-1β (Fig. 4B) levels when treated with CR-LAAO at 1.5 and 1.7 μg/mL. HepG2 cells treated with CR-LAAO also released increased levels of IL-6 at 10.78 μg/mL (Fig. 4C) and of IL-1β at all concentrations evaluated (Fig. 4D) when compared to non-stimulated cells. Taken together, these results suggest that the production of inflammatory mediators such as IL-6 and IL-1β may be related to the apoptosis promoted by CR-LAAO.

### CR-LAAO induces apoptosis in tumor cell lines via oxidative stress

Since CR-LAAO was cytotoxic to tumor cells^11^ and induced increased levels of IL-1β, our next step was to investigate the induction of apoptosis in tumor cells (HL-60 and HepG2), assessed by labeling with annexin V-FITC and PI. Cisplatin was used as control of cell death. The results were expressed by quantifying the percentages of cells labeled with annexin V+/PI+ and annexin V+/PI−.

**Figure 5:**
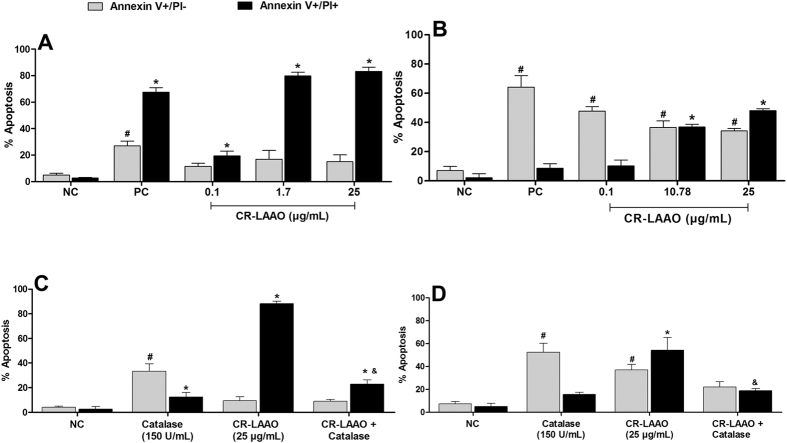
CR-LAAO induces apoptosis in tumor cells mediated by oxidative stress. HL-60 (**A**,**C**) and HepG2 (**B**,**D**) tumor cells were stimulated with CR-LAAO (0.1, 1.7, 10.78 or 25 μg/mL) in the absence or presence of catalase (150 U/mL) for 24 h. Non-stimulated culture cells were used as negative controls (NC). The positive control (PC) consisted of stimulation with cisplatin at concentrations previously determined by the MTT assay for each cell line (IC_50_ for HL-60: 2.9 μg/mL; HepG2: 4.0 μg/mL). Data were expressed as means ± SD of the percentage of annexin V+/PI− or annexin V+/PI+ stained cells (n = 4). ^#^p < 0.05 (PI+/AV− values in relation to NC); *p < 0.05 (PI+/AV+ values in relation to NC); & p < 0.05 (PI+/AV+ values in relation to PI+/AV+ of cells stimulated only with CR-LAAO).

CR-LAAO concentrations (0.1, 1.6 and 25 μg/mL) promoted HL-60 cell death by apoptosis with high percentages of cells marked with annexin V+/PI+ (19; 80 and 83%, respectively) (Fig. 5A). CR-LAAO concentration of 0.1 μg/mL increased the percentage of HepG2 cells labeled with annexin V+/PI− (48%), while the concentrations of 10.78 and 25 μg/mL induced cell apoptosis with increased percentages of cells labeled with annexin V+/PI+ (36 and 48%, respectively) (Fig. 5B).

Next, the involvement of hydrogen peroxide produced by CR-LAAO in the apoptosis process was evaluated by treating the cells with the toxin (25 μg/mL) together with catalase (150 U/mL). The results showed alterations in the apoptotic profile of CR-LAAO for both tumor cell lines, with reduction of approximately 60% and 30% of the apoptotic effect on HL-60 (Fig. 5C) and HepG2 (Fig. 5D) cells, respectively.

### CR-LAAO induces caspases activation in tumor cell lines

**Figure 6:**
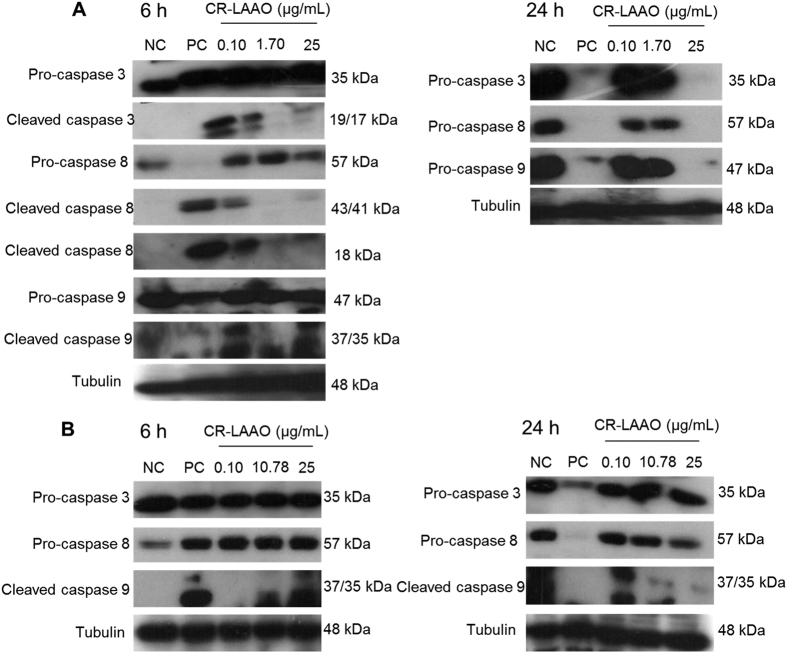
Western blot analysis of protein expression in HL-60 and HepG2 stimulated with CR-LAAO. Activation of caspases 3, 8 and 9 was detected after 6 h and 24 h of the stimulus of HL-60 (**A**) and HepG2 (**B**) cells with CR-LAAO. NC: negative control (non-stimulated cells); PC: positive control (cisplatin at concentrations previously determined by the MTT assay for each cell line: IC_50_ for HL-60: 2.9 μg/mL; HepG2: 4.0 μg/mL).

The CR-LAAO-induced activation of caspases on tumor cells was evaluated by Western blot in order to confirm the induced-apoptosis in HL-60 and HepG2 and to determine which apoptosis pathway (intrinsic or extrinsic) was activated in response to stimulation with the toxin. After 6 h of stimulus of HL-60 cells with CR-LAAO, cleaved forms of caspases 3, 8 and 9 could be observed, indicating the activation of apoptosis by both the intrinsic and extrinsic pathways (Fig. 6A). After 24 h of stimulus, we only observed reduction in pro-caspases 3, 8 and 9 at a CR-LAAO concentration of 25 μg/mL (Fig. 6B). Regarding HepG2 cells, a 6 h stimulation period with CR-LAAO increased the cleaved form of caspase 9 (Fig. 6C). After 24 h, pro-caspase 8 was decreased and the cleaved form of caspase 9 was increased by CR-LAAO, suggesting the activation of the extrinsic and intrinsic pathways of apoptosis (Fig. 6D).

### CR-LAAO and the expression of apoptotic genes in tumor cell lines

**Figure 7:**
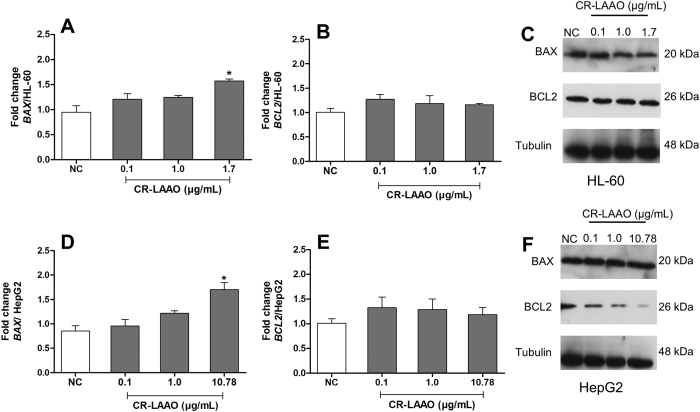
Expression profile of *BAX* and *BCL2* genes. The expression of *BAX* and *BCL2* genes was determined by quantitative reverse PCR. The results are presented as an average fold change in the expression of each gene in HL-60 (**A**,**B**) and HepG2 (**D**,**E**) cells stimulated with CR-LAAO (0.1; 1.0; 1.7 and 10.78 μg/mL). Non-stimulated culture cells were used as negative controls (NC). ACTB (β-actin) was used as a reference gene. Data were expressed as means ± SD (n = 3). *p < 0.05 compared to NC. The protein expression of *BAX* and *BCL2* genes was detected by Western blot after 24 h of the stimulus of HL-60 (**C**) and HepG2 (**F**) tumor cells with CR-LAAO (0.1; 1.0; 1.7 and 10.78 μg/mL). NC: negative control (non-stimulated cells).

The expression of genes involved in the apoptosis process (*BCL2* and *BAX*) by HepG2 and HL-60 tumor cells treated with CR-LAAO was evaluated by RT-qPCR. The pro-apoptotic gene *BAX* was up regulated with CR-LAAO concentrations of 1.7 and 10.78 μg/mL in HL-60 (Fig. 7A) and HepG2 cells (Fig. 7D), respectively, when compared to non-stimulated cells. However, the anti-apoptotic *BCL2* gene expression was not altered by any concentration tested (Fig. 7B and E). Then, protein expression of BCL2 and BAX was assessed by Western blot (Fig. 7C and F), which showed decreased expression of anti-apoptotic BCL2 protein by HepG2 cells treated with different concentrations of CR-LAAO (Fig. 7F).

### Cell cycle analysis

**Figure 8:**
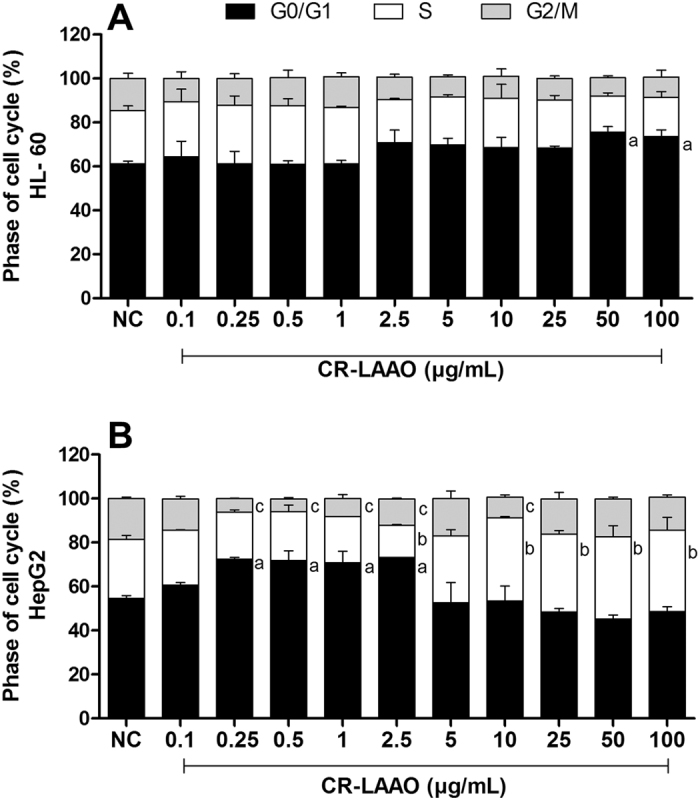
Cell cycle modulation induced in tumor cells by CR-LAAO. HL-60 (**A**) and HepG2 (**B**) tumor cells were stimulated with CR-LAAO (0.1–100 μg/mL). Non-stimulated cells were used as negative controls (NC). Cells were fixed with ethanol and stained with PI from HFS (Hypotonic Fluorescence Solution). G0/G1: rest/preparation for synthesis; S: DNA synthesis; G2/M: preparation for mitosis. Data were expressed as means ± SD (n = 4). ^a^p < 0.05 (G0/G1 values in relation to NC); ^b^p < 0.05 (S values in relation to NC) and ^c^p < 0.05 (G2/M values in relation to NC).

The cell cycle kinetic analysis was performed by flow cytometry in order to evaluate the effects of CR-LAAO in the process of cell division. HL-60 cells stimulated with CR-LAAO at the concentrations of 50 and 100 μg/mL showed different distribution in the phases of the cycle (G0/G1, S, G2/M) in comparison to non-stimulated cells, with significantly increased percentage of cells in the G0/G1 phase (Fig. 8A). In addition, the distribution of HepG2 cells in the three phases of the cell cycle revealed that CR-LAAO concentrations from 0.25 to 2.5 μg/mL were able to promote delay in the G0/G1 phase, while concentrations from 5 to 100 μg/mL induced delay in the S phase of the cell cycle (Fig. 8B).

## Discussion

The inflammatory process promoted by snake envenomations is mainly manifested locally at the site of the bite, and the key toxins involved are phospholipases A_2_^16^,^17^ and metalloproteases^18^,^19^, though other toxins have also been described with pro-inflammatory activity, such as serine proteases^20^ and LAAOs^12^.

LAAOs from snake venoms (SV-LAAOs) play important roles in various venom-induced biological effects, such as induction of platelet aggregation, apoptosis and bactericidal, fungicidal, antiparasitic and anti-HIV activities^8^,^9^,^11^,^21^,^22^. Several of these effects of SV-LAAOs have been mainly attributed to the release of hydrogen peroxide into the medium, resulting in oxidative stress and consequent cell death^23^. However, the effects of SV-LAAOs in the inflammatory response and its mechanisms of induced cell death are poorly described.

In the present study, we investigated the pro-inflammatory and pro-apoptotic effects of CR-LAAO, an L-amino acid oxidase isolated from *Calloselasma rhodostoma* venom. CR-LAAO was capable of inducing acute inflammatory responses *in vivo*, with neutrophil recruitment resulting in increased levels of pro-inflammatory cytokines (IL-6 and IL-1β) and lipid mediators (PGE_2_ and LTB_4_). *In vitro* analyses showed the ability of this toxin to activate peritoneal macrophages extracted from mice to produce IL-6 and IL-1β, which showed to be dependent of the activation of TLR2 and TLR4.

Similar to CR-LAAO, BF-LAAO from *Bungarus fasciatus* venom promoted a significant increase in the number of neutrophils in the peritoneal cavity of mice after 6 and 16 h of stimulus^12^. In acute inflammatory responses such as that induced by CR-LAAO, neutrophils migrate quickly to the injured site and are the main responsible for the initial phagocytosis of infectious agents and release of cytokines such as IL-1 and IL-6^24^.

Cytokines are produced by several types of immune cells. They influence the activity, differentiation, proliferation and survival of immune cells and regulate the production and activity of other cytokines, which may increase or attenuate the inflammatory process^25^. In the inflammatory response induced by CR-LAAO, no production of anti-inflammatory cytokines such as IL-4 or IL-10 was observed, which indicates that this toxin mainly induces pro-inflammatory effects.

Besides cytokines, CR-LAAO also induced the production of lipid mediators such as PGE_2_ and LTB_4_. During inflammation, prostaglandins (PGs) and leukotrienes (LTs) may be released in addition to cytokines by different activated cells, such as neutrophils, mast cells and macrophages. These mediators are produced after membrane disturbances that lead to increases in intracellular calcium^26^,^27^. Our results suggest that this production of lipid mediators should be related to the activation of neutrophils in the peritoneal cavity of mice, as well as the action of released pro-inflammatory cytokines.

Pontes *et al*.^13^,^15^ also showed that CR-LAAO was capable of activating human neutrophils *in vitro* to produce pro-inflammatory cytokines (IL-6, IL-8 and TNF-α) and lipid mediators (LTB_4_ e PGE_2_). Complementing those findings, our results suggest that CR-LAAO is responsible for triggering complex immunologic pathways for the production of such inflammatory mediators, initiating an acute inflammatory response dependent of toll-like receptors such as TLR2 and TLR4.

Toll-like receptors (TLRs) constitute a conserved family of receptors responsible for the recognition of pathogen-associated molecular patterns (PAMPs) and tissue damage signals (DAMPs) generated by injured cells and tissues^28^. Zoccal *et al*.^29^ presented the concept of venom-associated molecular patterns (VAMPs) to refer to molecules that are introduced into the host by stings and are recognized by pattern recognition receptors (PRRs), resulting in inflammation. In this context, we can suggest that CR-LAAO, like other venoms, acts activating these receptors and inducing the production of pro-inflammatory cytokines and co-stimulatory molecules that play important roles in the activation of inflammatory responses.

Considering CR-LAAO’s inflammatory effects and aiming at potential antitumor applications for this toxin, our next objectives were to investigate the possible relation between CR-LAAO-induced inflammation and its previous described cytotoxic effects on tumor cells (HL-60 and HepG2)^11^. First, we showed that CR-LAAO induced the release of significant levels of IL-6 and IL-1β after 6 h of stimulation of HL-60 and HepG2 tumor cells. The production of pro-inflammatory cytokines by these tumor cells could contribute to the recruitment of immune cells that would facilitate the combat of cancer. Then, to evaluate if the release of such cytokines could contribute to the high cytotoxicity of CR-LAAO on tumor cells, we investigated the pro-apoptotic potential of this enzyme by staining with annexin V-FITC and PI conjugated to fluorescein, which is a marker of phosphatidylserine exposed by cells at different stages of apoptosis^30^. The initial phase of apoptosis (annexin V+/PI−) is characterized by the preservation of membrane integrity, while in the late stage of apoptosis (annexin V+/PI+) there is a breakdown of the plasma membrane, triggering several events that can culminate in cell necrosis^31^,^32^.

CR-LAAO was able to induce apoptosis in HL-60 and HepG2 tumor cell lines after 24 h of stimulus. However, this effect was partially inhibited in the presence of catalase, suggesting that hydrogen peroxide should be related to the apoptosis induced by this toxin. Numerous studies have reported that apoptotic processes induced by SV-LAAOs are partly explained by the generation of hydrogen peroxide, which accumulates on the surface of cell membranes. Hydrogen peroxide is a reactive oxygen species (ROS), and is widely accepted that increased ROS concentrations promote cell mitochondrial breakdown, causing its death^8^,^21^,^23^,^33^,^34^,^35^. Nevertheless, the fact that catalase only partially inhibited the process of apoptosis induced by CR-LAAO reinforces the hypothesis that the activation of cells of the immune system as well as the production of pro-inflammatory cytokines may be involved in the induction of tumor cell death by this LAAO.

In addition, we evaluated the activation of pro-apoptotic caspases in the tumor cells stimulated with CR-LAAO. The apoptosis process induced by CR-LAAO in HL-60 and HepG2 tumor cells occurred by activation of the intrinsic and extrinsic pathways, with more evident activation of the intrinsic pathway for HepG2 cells. The fact that pro-caspase 3 was not activated by CR-LAAO could be related to insufficient stimulation periods, while the activation of caspase 8 and mainly of cleaved caspase 9 indicates that cells were in apoptosis process. This result confirms the data obtained by labeling with annexin, which detected a much smaller percentage of HepG2 cells in apoptosis (~40%) compared to HL-60 cells (~80%). This could be explained by possible sensitization of cells to the death process, considering that HepG2 cells proved to be more resistant to CR-LAAO than HL-60 cells^11^.

Other SV-LAAOs have been evaluated regarding the induction of apoptosis, showing similar effects to CR-LAAO, e.g. BatroxLAAO activated caspases 3 and 9 after 24 h of stimulus of PC12, HL-60, Jurkat and B16F10 cell lines, suggesting activation of the intrinsic pathway of apoptosis^34^; BpirLAAO activated caspases 3, 8 and 9 after 18 h of stimulus of HL-60 and HL-60.Bcr-Abl lines, suggesting activation of the intrinsic and extrinsic pathways of apoptosis^36^. The LAAO isolated from *O. hannah* venom was also able to activate the effector caspases 3 and 7^37^, endorsing the effectiveness of this class of enzymes in the activation of the apoptosis process.

Although we did not observe a difference in the levels of the BAX protein in Western blot analyses, expression of pro-apoptotic *BAX* gene by tumor cells stimulated with CR-LAAO further demonstrated the potential of this enzyme as an apoptosis inducer. Generally, tumor cells overexpress anti-apoptotic proteins and decrease the pro-apoptotic ones^31^, which significantly contributes to failure of cancer therapies. CR-LAAO was able to avoid this mechanism by promoting induction of apoptosis in cancer cells, which is the ideal strategy for the development of new therapies for cancer.

Once CR-LAAO-induced apoptosis was demonstrated on tumor cells, our next goal was to evaluate the influence of this toxin in the cell cycle modulation. Under normal conditions, the cell division process occurs continuously and repetitively. It is known that during cell cycle there are several checkpoints, i.e. signaling mechanisms that detect DNA damage. Cellular repair proteins control the inhibition or progression of the cycle through different pathways, promoting delays in phases of the cell cycle. The progression is resumed only when the damage is repaired, and cells with irreparable damage go into apoptosis. Tumor cells have mechanisms to evade these checkpoints, allowing rapid replication of these cells^38^.

CR-LAAO was able to interfere with the cell cycle of both tumor cell lines evaluated, delaying the G0/G1 phase of HL-60 cells, and G0/G1 and S phases of HepG2 cells. Although these observed delays could be somehow associated with the apoptosis described earlier, we could not see a direct correlation between these results, thus further studies should be carried out in the future for a better understanding of this matter. Nevertheless, a few other studies have shown similar effects for SV-LAAOs in the cell cycle modulation, e.g. BatroxLAAO and ACTX-6 also caused delay in G0/G1 phase of HL-60 and A549 tumor cells, respectively. These delays could prevent the initiation of DNA synthesis and consequently the replication of tumor cells^21^,^39^.

These results of cell cycle modulation, together with data on cytotoxicity^11^, apoptosis induction and pro-inflammatory effects of CR-LAAO, suggest that this toxin exhibits an excellent antitumor potential, which opens up prospects for the development of novel immunotherapeutic strategies against cancer.

In conclusion, CR-LAAO was able to induce acute inflammatory responses *in vivo*, with recruitment of neutrophils and increased levels of cytokines and lipid mediators. *In vitro*, it increased IL-6 and IL-1β production in macrophages associated with the activation of TLR2 and TLR4. Moreover, this toxin promoted apoptosis in HL-60 and HepG2 tumor cells mediated by oxidative stress and the activation of immune cells, resulting in caspase activation and expression of the pro-apoptotic gene *BAX*. Our findings may open new paths of research and we speculate that the activation of immune cells can potentiate the antitumor effects of this class of toxins.

## Materials and Methods

### Toxin

CR-LAAO was purified from *Calloselasma rhodostoma* snake venom as previously described^14^ and kindly donated by Prof. Dr. Sandro Ghisla from University of Konstanz, Germany. The lyophilized protein was stored at 4 °C and solubilized in PBS (phosphate buffered saline) immediately before its use in each experiment. Total protein concentration was determined by the microbiuret assay^40^. Before performing any biological assay, the L-amino acid oxidase specific activity test was determined by a spectrophotometric assay using L-leucine as substrate^41^, in order to assure the enzymatic activity of CR-LAAO.

### Animals

Male BALB/c and C57BL/6 (wild type, TLR2 and TLR4 knockouts) mice (6-8 weeks old) were obtained from the animal facilities of the Faculdade de Ciências Farmacêuticas de Ribeirão Preto (Universidade de São Paulo, Ribeirão Preto, Brazil). Animal care procedures were performed according to the Brazilian College of Animal Experimentation (COBEA) guidelines and the experimental protocols were approved by the Committee for Ethics on Animal Use (CEUA) from FCFRP-USP (Register n° 11.1.497.53.6).

### Cell lines

The following tumor cell lines were obtained from ATCC (American Type Culture Collection, Rockville, MD, USA):

- HL-60 (ATCC CCL-240) – human promyelocytic leukemia
- HepG2 (ATCC HB-8065) – human liver carcinoma

HL-60 cells were cultured in complete RPMI (Roswell Park Memorial Institute) 1640 medium and HepG2 cells were cultured in complete DMEM (Dulbecco’s Modified Eagle Medium), and both were supplemented with 10% fetal bovine serum and 1% penicillin/streptomycin, under an atmosphere of 5% CO_2_ and 95% air, at 37 °C.

### Inflammatory analyses

#### Induction of peritoneal inflammatory reaction

Group of four BALB/c mice were injected intraperitoneally (i.p.) with 300 μL of sterile PBS (negative control) or CR-LAAO at different concentrations (1–50 μg/mL in PBS). After 4 h of stimulus, the animals were euthanized by instillation of CO_2_ and their peritoneal cavities were washed with 3 mL of cold PBS, draining the exudate aseptically with a syringe. The total leukocyte count was performed in a Neubauer chamber, analyzing each exudate sample diluted in Turk’s solution (1:20).

Then, a CR-LAAO concentration was fixed (25 μg/mL in PBS) and different stimulation periods (4, 24, 48 and 96 h) were investigated. Injection, euthanasia and total leukocyte counts were performed as described above. The differential leukocyte count in neutrophils or mononuclear cells was done by concentrating the different exudate samples on microscope slides using Cytospin (Shandon Souther Products Ltd., Manchester, UK), followed by staining with Romanowsky stain and examination under an optical microscope (Bioval, São Paulo, Brazil) at magnification of 400X. One hundred cells were counted per slide. After centrifugation (400 × g, for 10 min, at 10 °C), the cell free peritoneal fluid was used to measure the levels of inflammatory mediators.

### Quantification of mediators

#### Cytokine measurements

The cell free peritoneal fluid obtained from mice injected with the toxin was used to measure TNF-α, IL-1β, IL-4, IL-6, and IL-10 by ELISA using specific antibodies (purified and biotinylated) and cytokine standards, according to the manufacturer’s instructions (R&D Systems, Minneapolis, USA). Optical densities were measured at 405 nm in a microplate reader (μQuant, Biotek Instruments Inc.). For each sample, cytokine levels were obtained from a standard curve established with the appropriate recombinant cytokine, and results were expressed in pg/mL. The detection limit was >10 pg/mL.

#### Eicosanoid measurements

LTB_4_ and PGE_2_ levels were measured in the cell free peritoneal fluid obtained from mice injected with the toxin, using specific enzyme immunoassays (Cayman Chemical) according to the manufacturer’s instructions. The sample absorbance was measured at 420 nm in a micro plate reader (μQuant, Biotek Instruments Inc.), and the concentrations of eicosanoids were calculated based on the standard curve. The detection limit was >13 pg/ml.

#### Macrophage culture and activation

The macrophages were isolated from the peritoneal cavities of C57BL/6 wild type or knockout (TLR2^−/−^, TLR4^−/−^) mice and plated in 96-well micro culture plates at a density of 2 × 10^5^ cells/well in RPMI medium supplemented with 10 mM L-glutamine, 100 U/ml penicillin, 100 U/ml streptomycin and 10% fetal bovine serum (FBS). The cells were cultured at 37 °C in a humidified 5% CO_2_ atmosphere for 18 h. After this period, the supernatants were collected and the cells were stimulated with CR-LAAO (0.19; 0.38, 0.75 and 1.5 μg/mL per well) for 24 h in RPMI without FBS at 37 °C in a 5% CO_2_ atmosphere. Non-stimulated culture cells were used as negative controls. After the stimulation period, the culture supernatants were collected to determine the IL-6 and IL-1β levels by ELISA, as described above.

#### Inflammatory mediators in supernatants of tumor cells

HL-60 and HepG2 tumor cells (2 × 10^5^ cells/well) were treated with different concentrations of CR-LAAO (0.75; 1.5; 1.7 and 10.78 μg/ml) for 6 h. Non-stimulated culture cells were used as negative controls. After the stimulation period, the culture supernatants were collected for IL-6 and IL-1β measurement by ELISA, as described above. The concentrations of 1.7 and 10.78 μg/ml represent the IC_50_ values of HL-60 and HepG2, respectively, which were previously determined by the MTT assay for each cell line^11^.

#### Apoptosis analyses

##### Flow cytometry analysis

Phosphatidyl serine exposure was determined by flow cytometry using the Dead Cell Apoptosis Kit with Annexin V FITC and propidium iodide (PI) (Molecular Probes™). Cultures of HL-60 and HepG2 tumor cells were treated with CR-LAAO (0.1; 1.7, 10.78 or 25 μg/mL) for 24 h. Non-stimulated culture cells were used as negative controls, and the positive controls received cisplatin (Incel, Darrow®) at concentrations previously determined by the MTT assay for each cell line (IC_50_ for HepG2: 4.0 μg/mL; HL-60: 2.9 μg/mL)^11^. Tumor cells were also treated with CR-LAAO (25 μg/mL) together with catalase (150 U/mL). Cells were washed twice with PBS, resuspended in a working solution containing PI (5 μg/mL) and annexin V (0.25 μg/mL), incubated for 15 min at 4 °C, and analyzed in a FACSCanto flow cytometer (Becton Dickison, Franklin Lakes, NJ, USA) using the Diva software. Approximately 1 × 10^6^ cells were analyzed for each treatment.

#### Western blot analysis of protein expression

HL-60 and HepG2 tumor cells (1 × 10^6^ cells) were stimulated with CR-LAAO (0.1; 1.7; 10.78 and 25 μg/mL) for 6 or 24 h at 37 °C and under an atmosphere of 5% CO_2_. Non-stimulated cells (negative control) were cultured under the same conditions. Afterward, the cells were collected and suspended in the western blot lysis buffer (20 mM Tris-HCl pH 7.4, 150 mM NaCl, 1 mM EDTA and phosphatase and protease inhibitors). Total protein concentration in the samples was determined using the BCA protein assay reagent, according to the manufacturer’s instructions (Thermo Fischer Scientific, Waltham, MA, USA). To separate the proteins of interest according to their molecular weight, equal amounts of protein were analyzed by 12% SDS-PAGE, and transferred onto polyvinylidene difluoride (PVDF) membranes (Amersham, GE Healthcare Life Science, Pittsburgh, PA, USA). The membranes were blocked for 2 h with 5% non-fat dry milk prepared in TBS-Tween (20 mM Tris, 137 mM NaCl, 0.01% Tween 20). Next, the PVDF membranes were incubated overnight with the following primary antibodies: anti-caspase 3 (code 96625, Cell Signaling), anti-caspase 8 (code 9746, Cell Signaling), anti-caspase 9 (code 9502, Cell Signaling), anti-BAX (code 2772, Cell Signaling), anti-BCL2 (code 2870, Cell Signaling) and anti-tubulin (code T3320, Sigma-Aldrich, St. Louis, MO, USA). Afterward, the membranes were incubated with the appropriate secondary antibodies and the proteins of interest were revealed by ECL (Amersham, GE Healthcare Life Science, Pittsburgh, PA, USA). The protein tubulin was used as an internal standard to normalize protein load among samples.

### RNA preparation and quantitative reverse transcription polymerase chain reaction (RT-qPCR)

#### RNA isolation and purification

Initially, HL-60 and HepG2 tumor cells lines (3 × 10^6^ cells) were treated with CR-LAAO at different concentrations (0.1, 1.0, 1.7 and 10.78 μg/mL). Non-stimulated culture cells were used as negative controls. After 24 h of treatment, cells were harvested and processed in accordance with the guidelines of the RNA extraction kit (SV Total RNA Isolation System, Promega Z3100). Three extractions (experiments) were performed independently in triplicate (n = 9). The integrity of the RNA was determined using the 18 S and 28 S rRNA bands following electrophoresis on a denaturing agarose gel. RNA concentrations and quality were measured using a spectrophotometer (NanoDrop spectrophotomer ND-1000), and all samples complied with the pre-established ratios (A260/A280 and A260/A230).

#### Synthesis of cDNA and RT-qPCR

After quantification and evaluation of the integrity of the obtained RNAs, the samples were processed for synthesis of cDNAs by *High Capacity cDNA Reverse Transcription* kit (Applied Biosystems cat #4368814), which were used in the real-time PCR assays for the quantification of mRNA of genes of interest in StepOnePlus™ equipment (Applied Biosystems, Carlsbad, CA, USA). To this end, specific TaqMan® probes for real-time PCR were acquired for amplification of *BAX* and *BCL2* genes. Two housekeeping genes were used for normalization: β-actin (*ACTB*) and glyceraldehyde-3-phosphate dehydrogenase (*GAPDH*).

Relative gene expression were assessed by comparing the values of cycle threshold (Ct) between treated groups and the respective negative controls by using 2^−ΔΔCt^ method^42^.

#### Cell cycle analysis

The assay was carried out according to previously described method^21^. HepG2 and HL-60 cells were treated with CR-LAAO (0.1; 0.25; 0.5; 1; 2.5; 5; 10; 25; 50 and 100 μg/mL) for 24 h. Non-stimulated culture cells were used as negative controls. After the stimulation period, the cells were treated with RNase-A (50 U/mL) and labeled with PI (20 μg/ml) for 3 h at room temperature. The percentage of cells in each phase of the cell cycle was determined by flow cytometry in a FACSCanto (Becton Dickison, Franklin Lakes, NJ, USA) and analyzed using the ModFit LT 2.0 1995-6 software (Verity Software House Inc.).

#### Statistical analysis

Statistical analysis of the results was performed by *GraphPad Prism 5* software, using one-way ANOVA method and Tukey post-tests with p < 0.05, comparing all treatments to the negative controls.

## Additional Information

**How to cite this article:** Costa, T. R. *et al*. CR-LAAO, an L-amino acid oxidase from *Calloselasma rhodostoma* venom, as a potential tool for developing novel immunotherapeutic strategies against cancer. *Sci. Rep.* **7**, 42673; doi: 10.1038/srep42673 (2017).

**Publisher's note:** Springer Nature remains neutral with regard to jurisdictional claims in published maps and institutional affiliations.
